# Sheep placental extract ameliorates polycystic ovary syndrome in rats by regulating ERβ-mediated Treg cells via the TGF-β1/Smad3 signaling pathway

**DOI:** 10.3389/fimmu.2026.1777841

**Published:** 2026-04-13

**Authors:** Cunlin Zhang, Zhijia Yan, Xinmeng Xing, Yanan Tang, Liru Jia, Xingrong He, Junbai Ma, Xiaoyan Bai, Hao Wang, Yuan Lin, Xiaoxia Zhang

**Affiliations:** 1 Department of Pathogenic Biology and Medical Immunology, Ningxia Key Laboratory of Infection and Immunity, School of Basic Medical Sciences, Ningxia Medical University, Yinchuan, China; 2 College of Labratory Medicine, Ningxia Medical University, Yinchuan, China; 3 Yinchuan Yibaisheng Bio-engineering Co., Ltd, Yinchuan, China; 4 College of Laboratory Medicine, Ningxia Medical University, Yinchuan, China; 5 College of Traditional Chinese Medicine, Ningxia Medical University, Yinchuan, China

**Keywords:** anti-inflammation, estrogen receptor β, gut dysbiosis, polycystic ovary syndrome, regulatory T cells, sheep placenta extract

## Abstract

Polycystic ovary syndrome (PCOS), is a common endocrine disorder associated with chronic inflammation, affecting reproductive health in women. Chronic inflammation is a core driver of PCOS, highlighting the urgent need for interventions that target the underlying inflammatory pathogenesis of PCOS. Sheep placenta extract (SPE), as a traditional medicinal substance with multiple biological effects, including anti-inflammation, has been suggested to offer potential therapeutic benefits in managing chronic inflammation associated with PCOS. Treg dysfunction has emerged as a critical role in many diseases related to chronic inflammation. However, the role of SPE and its anti-inflammation mechanisms related to Treg in PCOS remains underexplored. The gut microbiota (GM) exerts a pivotal role in the pathogenesis and progression of numerous inflammatory disorders. Accordingly, further research is needed to investigate how SPE influences the gut microbial composition. The PCOS model was induced in rats using letrozole, and the animals were then administered SPE. Inflammatory cytokine, sex steroid hormone levels, markers of metabolic, transcriptomics, and 16S rRNA sequencing were assessed. SPE significantly alleviated abnormal ovarian histopathology in PCOS rats. SPE treatment suppressed inflammation by upregulating regulatory T cells (Tregs), with their anti-inflammatory effect mediated through the ERβ-TGFβ1-Smad3 signaling pathway. SPE may ameliorate pathological damage in ovarian tissue of PCOS rats by exerting anti-inflammatory effects through the estrogen receptor β (ERβ)/regulatory T cell (Treg) axis mediated by TGF-β1/Smad3 signaling, alonged with rectifying gut dysbiosis. Further study will focus on the proteomics strategy for identifying SPE, larger-scale validation of samples and comparisons with metformin or GLP-1 receptor agonists.

## 1 Introduction

Polycystic ovary syndrome (PCOS) is a common endocrine disorder affecting 5-21% of reproductive-aged women worldwide, characterized by hyperandrogenism, ovulatory dysfunction, and polycystic ovarian morphology (1–3). In addition, PCOS significantly elevates the risk of developing several conditions, including metabolic disorders such as type 2 diabetes, cardiovascular disease, metabolic syndrome, endometrial cancer, infertility, obstetrical complications, and psychiatric dis-orders (4–7). Growing evidence indicates that chronic inflammation serves as a key pathological mechanism underlying both the reproductive and metabolic features of PCOS (8). However, the exact role related to chronic inflammation in PCOS is still unclear.

Current therapeutic approaches for PCOS primarily focus on managing symptoms rather than addressing the underlying inflammatory pathogenesis. Conventional treatments, including oral contraceptives, insulin sensitizers and anti-androgens, are only partially effective and have adverse effects with long-term use (9). This therapeutic gap highlights the urgent need for new interventions that specifically target inflammatory pathways in PCOS. Traditional Chinese medicine offers promising alternatives for managing PCOS. One such alternative is sheep placenta extract (SPE), which contains multiple bioactive components with demonstrated anti-inflammatory, immunomodulatory and antioxidant properties (10, 11). However, the anti-inflammatory effect of SPE and underlying mechanisms related to immune-regulation on PCOS need to be explored.

Emerging studies indicated that chronic inflammation plays a pivotal role in the pathogenesis of various chronic disorders, including PCOS (12). A dysregulation of immune cells and inflammatory cytokines is evident in the serum, ovaries, and other organs of patients with PCOS (13). The interplay between inflammatory state and obesity, HA and IR, leads to increased metabolic imbalance and reproductive-endocrine dysfunction in PCOS patients (12). Additionally, elevated levels of inflammatory factors, such as tumor necrosis factorα(TNF-α), interleukin-1β (IL-1β), interleukin-6 (IL-6), and inter-leukin-17A (IL-17A) are detected in the peripheral blood of PCOS patients, while anti-inflammatory cytokines including interleukin-10 (IL-10) and transforming growth factor-β(TGFβ) are reduced (13). Furthermore, chronic inflammation contributes to PCOS-related complications of multi-organ dysfunction, including cardiovascular diseases, and non-alcoholic fatty liver disease (14, 15). Therefore, a comprehensive understanding of chronic inflammation mediated by SPE in PCOS is crucial for effective prevention and management strategies.

During chronic inflammation in the endocrine and metabolic dysregulation of PCOS, studies demonstrated that T lymphocyte overactivation is a key driver (16, 17). Specifically, compared with age-matched healthy women, PCOS patients exhibit significantly higher numbers of immune cells in their peripheral blood, including lymphocytes, neutrophils, monocytes, and macrophages, as well as elevated macrophage and lymphocyte infiltration in their ovaries (18, 19). As a core component of the adaptive immune system, T lymphocytes maintain balance between pathogen defense and self-tolerance (20). Among these, Tregs, a specialized subset of CD4^+^ T cells suppress excessive inflammation responses to prevent autoimmune diseases and chronic inflammation (21). In the peripheral environment, naive CD4^+^ T cells differentiate into Tregs via the TGF-β1-Smad signaling pathway (22). Studies also demonstrated the critical role of Tregs in modulating inflammation and immune regulation in chronic inflammatory and autoimmune diseases (23, 24). Moreover, studies showed that immune cells play pivotal role in the regulation of tissue repair and regeneration (25). Consequently, Treg dysfunction often triggers uncontrolled inflammation, high-lighting their indispensable role in maintaining immune homeostasis (24).

As a pivotal sex hormone, estradiol (E2) regulates the development of the reproductive system and profoundly influences adaptive immune homeostasis via its nuclear receptors, including estrogen receptor alpha (ERα) and beta (ERβ), which modulate Tregs differentiation, maintenance, and suppressive function (26). Furthermore, Tregs play a critical role in preventing autoimmunity and chronic inflammation via the ERα/ERβ signaling network. Notably, ERβ primarily promotes Treg development and stability through direct transcriptional regulation (27), while functionally sustaining their immunosuppressive activity by enhancing TGF-β and IL-10 secretion and dose-dependently inhibiting effector T cell proliferation and pro-inflammatory cytokines production (28). For instance, in a pneumonia model, E2 activates Treg function via ERβ signaling to suppress macrophage pro-inflammatory cascades and expedite pulmonary inflammation resolution and tissue repair (28). Although PCOS is a chronic inflammatory disorder closely associated with E2 levels and receptor expression (29), the exact role and underlying mechanism by which E2-ER interaction regulates Tregs for anti-inflammation in PCOS need to be further investigated.

In recent years, the gut microbiota (GM) and associated metabolic dysregulation have been extensively implicated in the pathogenesis of chronic diseases, including PCOS (30). Emerging evidence underscores the pivotal role of GM dysbiosis in chronic inflammation, insulin resistance, hyperandrogenism, and metabolic syndrome, thereby contributing to PCOS etiology through modulation of energy homeostasis, short-chain fatty acid (SCFA) pathway, lipopolysaccharide (LPS)-mediated endotoxemia, bile acid (BA) metabolism, and intestinal barrier integrity (31–33). Moreover, GM-derived metabolites, including SCFAs and secondary BAs, exert immunomodulatory effects by regulating the differentiation and function of immune cells, (e.g., Tregs, macrophages) and modulating cytokine profiles (e.g., IL-6, TNF-α, IL-1β) in the gut mucosa and systemic circulation (34). This interplay highlights the potential of GM as a therapeutic target for mitigating inflammatory responses associated with immune regulation in PCOS. However, little research has been conducted into the gut microbial composition and the related effects of SPE.

The present study aimed to assess the anti-inflammatory effects of SPE and further to elucidate mechanism related to anti-inflammation in PCOS. Additionally, this study was designed to investigate the impact of SPE on gut microbial composition.

## 2 Materials and methods

### 2.1 Animals, diet and intervening substance

Six-week-old specific-pathogen-free (SPF) female Sprague–Dawley (SD) rats (body weight, 180 ± 10 g) were purchased from the Laboratory Animal Center of Ningxia Medical University. and all rats were housed under specific pathogen-free (SPF) conditions at the Laboratory Animal Center of Ningxia Medical University, with controlled temperature (23 °C ± 5 °C), humidity (65% ± 5%), and a 12-h light/dark cycle (lights on from 08:00 to 20:00). Animals had free access to food and water. All rats were fed a commercial diet (46.65% crude protein, 20.73% moisture, 0.09% crude fat, 0.13% crude ash, 0.07% crude fiber, calcium, and phosphorus) provided by Keaoxieli Feed Co., Ltd. (Beijing, Chi-na). SPE was purchased from Yinchuan Yibaisheng Biological Engineering Co., Ltd. (Ningxia, China). Letrozole was obtained from Hengrui Pharmaceutical Co., Ltd. (Jiangsu, China). All experimental procedures complied with Chinese and international guidelines and were approved by the Ethics Committee of Ningxia Medical University (No.2024-3343).

### 2.2 Animal experimental design

**Figure 1:**
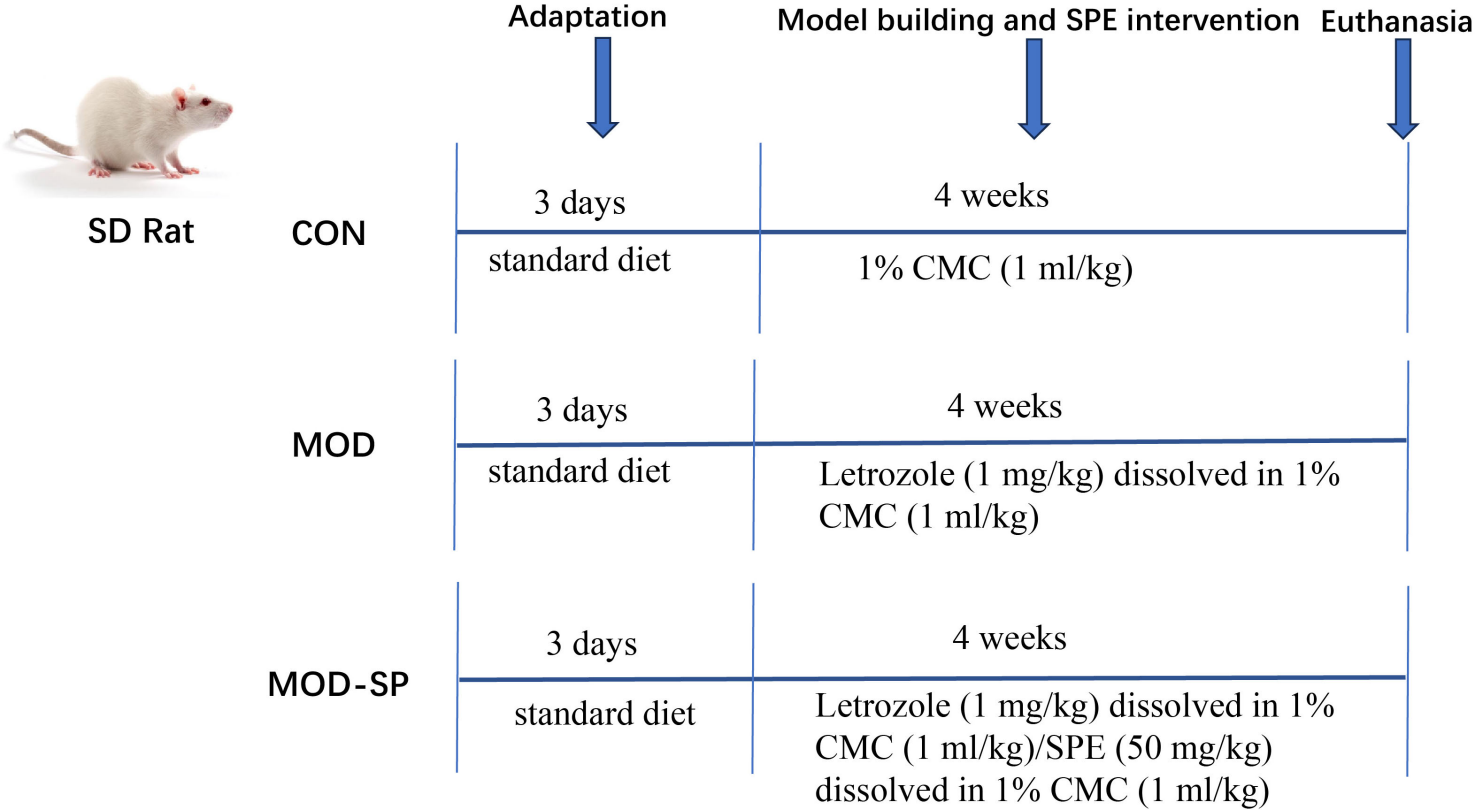
Schematic timeline of the animal experimental design. SD, Sprague-Dawley rats; CON, pair-fed control group; MOD, letrozole-induced PCOS, polycystic ovary syndrome model group; MOD-SP, SPE, sheep placental extract -supplemented PCOS model group.

The schematic timeline of the experimental design is shown in **Figure 1** Briefly, after a 3-day acclimation period with the control diet, 24 female SD rats (6-week-old) were randomly divided into three groups (n = 8 per group): (a) pair-fed control group (CON), (b) letrozole-induced PCOS model group (MOD), and (c) SPE-fed MOD group (MOD-SP). To eliminate confounding effects of differential food intake, pair-feeding was employed. Daily food consumption in the CON group (fed standard chow) was quantified individually, and the mean intake was calculated. On the subsequent day, equivalent amounts of the respective diets were provided to the MOD and MOD-SP groups. Food intake was monitored daily throughout the experimental period and body weights were recorded weekly. Rats in the MOD groups received 1 mg/kg letrozole daily via oral gavage for 4 weeks, dissolved in 1% carboxymethylcellulose (CMC) aqueous solution, corresponding to a solution volume concentration of 1 mL/kg. The solution volume was adjusted according to the animals’ body weight; while rats in the CON group received 1 mL/kg 1% CMC solution daily for 4 weeks. The establishment of the PCOS rat model was comparable to that of a previous description (35). In this study, the experimental intervention was administered at the same time as the induction of the disease model. The MOD-SP group received 1mg/kg letrozole daily alongside by 50 mg/kg SPE (11) (dissolved in 1% CMC aqueous solution) for 4 weeks. Body weight was recorded every 5 days for 28 days in all groups. At the endpoint of experiment, followed previous study (36), all rats were euthanized after intraperitoneal injection35 of 2% pentobarbital sodium at a dose of 200 mg/kg body weight, and relevant parameters were analyzed. Fecal samples were snap-frozen and stored at -80°C. Plasma samples were centrifuged and stored at -80°C.

### 2.3 SPE component identification

#### 2.3.1 SDS-PAGE analysis

A 10% separating gel were employed(Catalog No.: G2043-50T, Servicebio, Wu-han, China). The placental protein and protein hydrolysates were analyzed under non- reducing conditions. The gels were run at 80 V until the samples and the pre-stained Marker were separated and reached the bottom of the separation gel, and SDS-PAGE Coomassie Brilliant Blue staining was carried out as previously described (37).

#### 2.3.2 Nano LC-MS/MS analysis

1000μL of 75% methanol-water solution and 200 μL wash buffer were used to prior reduction lyophilized peptides before using Nano Liquid Chromatography-Tandem Mass Spectrometry (LC- MS/MS) to identify the sequence of peptide. After desalting and lyophilization, peptides were homo-dispersed in 20 μL of 0.1% formic acid for sequencing. Peptide sequencing was conducted by Shanghai Baiqu Biomedical Technology Co., Ltd. (Shanghai, China). The raw MS files were analyzed and searched in the Ovis aries protein database using Spectro Mine software (4.2.230428.52329). Parameters set: Carbamidomethyl [C] as a fixed modification, Oxidation (M) and Acetyl (Protein N-term) as variable modifications. No-Enzyme (Unspecific) was used as enzyme. A maximum of 2 missed cleavage(s) was allowed. The false discovery rate (FDR) was set to 0.01 for both PSM and peptide levels. Peptide identification was performed with an initial precursor mass deviation of up to 20 ppm and a fragment mass deviation of 20 ppm. All the other parameters were reserved as default. For the subsequent identification analysis, only highly confidently identified peptides were selected.

### 2.4 Body weight, food, and vaginal smear

During the experimental period, body weight was recorded every 3 days, the amount of food was recorded every 3 days, and the complete estrous cycle of rats of 4–5 days was monitored. Vaginal smears of rats were collected daily at 9 am for 5 days before euthanasia, and the stage of the estrous cycle was determined by microscopic observation of the main cell types, as previously described (38).

### 2.5 Ovary hematoxylin and eosin staining

After sacrificing the rats, ovarian tissues were immediately fixed in 4% paraformaldehyde, embedded in paraffin, and sectioned at 4 µm. To evaluate ovarian damage, every 10th section (n = 8 per group) was mounted on glass slides, stained with hematoxylin and eosin (H&E), and analyzed under an Olympus light microscope (Melville, NY, USA) by two observers blinded to the experimental groups. The numbers of cystic follicles and corpora lutea were quantified. Based on H&E-stained ovarian sections, the number of cystic follicles and corpora lutea, as well as the area of three ovarian tissue sections, were measured in each group. Data were normalized to the average ovarian tissue area in the negative control group. Cystic follicles were defined according to previously established criteria: follicles lacking oocytes, with a dilated antrum, thickened thecal cell layer, and a thin granulosa cell layer containing morphologically intact cells.

### 2.6 Blood glucose testing

After 8 hours of fasting, the FPG of one drop of blood was measured from the tail blood using a OneTouch Profile blood glucose meter (Johnson & Johnson, Inc., Milpitas, CA, USA).

### 2.7 Determination of plasma sex steroid hormones

Sex hormone levels were assessed by using enzyme-linked immunosorbent assay (ELISA) kits that were purchased from Shanghai Jianglai and strictly followed the instructions to detect follicle stimulating hormone (FSH), prolactin (PRL), progesterone (PG), estradiol (E2), testosterone (T), and luteinizing hormone (LH). All samples were analyzed in triplicate.

### 2.8 Plasma lipid metabolism

The biochemical indications of lipid metabolism, including plasma total cholesterol (TC), triglyceride (TG), high-density lipoprotein cholesterol (HDL-C), and low-density lipoprotein cholesterol (LDL-C), aspartate transaminase (AST) were respectively determined using Chemray 240 automatic biochemical analyzer (Shenzhen Radu Life Sciences, Shenzhen, China). Lipid levels were determined via an enzymatic colorimetric assay following the manufacturer’s instructions.

### 2.9 Determination of plasma indicators

Plasma inflammatory cytokines including IL-6 (Catalog No.: KE20024, Wuhan Tri-Eagle Biotechnology Co, Wuhan, China), IL-10 (Catalog No.: KE20003, Wuhan Tri-Eagle Biotechnology Co, Wuhan, China), IL-17A (Catalog No.: JL20880-96T, Shanghai Jianglai Biotechnology Co, Shanghai, China), TNF-α (Catalog No.: KE20018, Wuhan Tri-Eagle Biotechnology Co, Wuhan, China), and IL-1β (Catalog No.: JL20884-96T, Shanghai Jianglai Biotechnology Co, Shanghai, China) were measured by using ELISA kits according to the manufacturer’s instructions. The sensitivities of the assays were 0.1, 0.1, 0.1, and 0.1pg/mL for IL-6, IL-10, IL-17A, TNF-α, respectively. Each sample was tested in triplicate. The plate was incubated at 37 °C for 30 min. Then, 100 µL of the chromogenic substrate warmed to 37°C was added to each well, and incubation was extended for an additional 60 min at 37°C. The reaction was stopped by adding 100 µL of 25% solution of glacial acetic acid. Optical density at 450 nm measured with a microplate reader (Thermo Scientific, USA).

### 2.10 Staining and flow cytometry analysis

Multicolor flow cytometry was performed using a Cyto FLEX flow cytometer (Beckman Coulter, United States). Single viable cells were sequentially gated as follows: singlets were selected based on forward scatter area (FSC-A) versus height (FSC-H), followed by exclusion of dead cells. Lymphocytes were identified by forward and side scatter characteristics. Within the live lymphocyte population, CD4^+^ T cells were gated, and regulatory T cells (Tregs) were defined as CD4^+^Foxp3^+^. The gate for Estrogen receptor (alpha or beta) expression was subsequently set on Foxp3 X ER (alpha or beta) dot plots, and the percentage of positive cells was reported. The lymphocytes were stained with fluorescence-conjugated monoclonal antibodies as follows: FITC-anti-Rat CD4 (eBioscience, 11-0040-82, United States), PE-anti-Rat Foxp3 (eBioscience, 2176028, United States), PerCP-anti- Mouse Monoclonal ER beta (Novus, NBP2-33066PCP, United States), and APC-anti-Rat Estrogen Receptor alpha (SantaCruz, sc-53492AF647, United States), according to the manufacturer’s instructions. In addition, before staining cells with Foxp3 flow cytometry antibody, the Transcription Factor Buffer Set (BD Biosciences, 562574, United States) was used to immobilize and permeabilize of cells. To standardize the flow cytometric readouts across time, application settings were applied in each experiment. Data were analyzed using FlowJo software (FlowJo LLC, Ashland, OR, USA).

### 2.11 Quantitative real-time PCR

RNA was extracted from frozen ovarian tissue with a commercial RNA isolation kit, strictly following the manufacturer’s instructions. cDNA synthesis was performed through reverse transcription with a reverse transcriptase kit (Trans Gen Biotech, China). For each sample, raw cycle threshold (Ct) values of target genes were normalized to the reference gene GAPDH by computing ΔCt = Ct_-_target_-_ Ct _-_GAPDH. Relative expression levels were quantified via the2^^-ΔΔCt^ method, with ΔΔCt = ΔCt_-_sample - mean ΔCt-control, normalizing the control group mean to 1.0. All experiments were conducted in triplicate. The primer sequences applied for qPCR were outlined in **Supplementary Table 1**.

### 2.12 Western blot

Protein extracted from ovarian tissues and cells of different groups was resolved using sodium dodecyl sulfate-polyacrylamide gel electrophoresis and transferred to polyvinylidene fluoride membranes. After blocking, the membranes were incubated overnight with primary antibodies including TGFβ1(Catalog No.: 26155-1-AP, 1:1000 dilution, Proteintech, China), Smad3(Catalog No.: abs124584, 1:1000 dilution, Absin, China), p-Smad3 (Catalog No.: abs173912, 1:1000 dilution, Absin, China). The protein expression level of the β-actin (Catalog No.: 20536-1-AP, 1:1000 dilution, proteintech, China) was used as an internal standard, respectively. On the following day, the specimens were incubated with HRP-conjugated Goat anti-Rabbit lgG(H+L) (Catalog No.: RGAR001, 1:6000 dilution, proteintech, China) for 2 h at room temperature and visualized using the enhanced ECL reagent. The protein bands were quantified using the Image-J software. For each sample, target protein intensities were normalized to β-actin by calculating the ratio (target/β-actin). Relative protein expression was then determined as fold change relative to the mean normalized ratio of the control group, set to 1.0. All blots performed in triplicate.

### 2.13 Gut microbiota sequencing analysis

After 4 weeks of treatment, eight rats per group were randomly selected and housed in sterilized cages. Fresh fecal samples were collected and immediately stored at -80 °C for subsequent DNA extract. Briefly, rats were placed in clean cages with sterile filter paper, and gentle pressure was applied to the tail base and rectum to stimulate defecation. Fecal pellets were collected with sterile forceps into pre-chilled Eppendorf tubes and flash-frozen at -80 °C. Bacterial DNA extract was performed using cetyltrimethylammonium bromide (CTAB). Samples were homogenized with 1000 μL CTAB lysis buffer containing lysozyme (1mg/mL), incubated at 65 °C with intermittent vortexing, and centrifuged at 12,000 ×g for 10 min. The supernatant was mixed with phenol: chloroform: isoamyl alcohol (25:24:1, v/v), followed by phase separation centrifugation. This step was repeated with chloroform: isoamyl alcohol (24:1, v/v). DNA was precipitated from the aqueous phase by adding 0.7 volumes of isopropanol and incubating at -20 °C for 1 h. After centrifugation, the pellet was washed twice with 1 mL 75% ethanol, air-dried, and resuspended in ddH_2_O. Residual RNA was removed by incubation with 1 μL RNase A (10 mg/mL) at 37 °C for 15 min. Purified DNA was stored at -20 °C until analysis. The V3-V4 hypervariable regions of the 16S rRNA gene were amplified using Phusion^®^ High-Fidelity PCR Master Mix with GC Buffer (New England Biolabs, USA) with primers 341F (5’-CCTAYGGGRBGCASCAG-3) and 806R (5’-GGACTACNNGGGTATCTAAT-3’). PCR products were electrophoresed on 2% agarose gels and purified using the GeneJET Gel Extract Kit (Thermo Scientific, USA). Sequencing libraries were prepared with the TruSeq^®^ DNA PCR-Free Library Preparation Kit (Illumina, USA), quantified via Qubit fluorometry, and finally sequenced on an Illumina HiSeq 2500 platform (Novogene Bioinformatics Technology Co., Ltd., Beijing, China).

### 2.14 RNA sequencing

Total RNA was extracted from the ovary using TRIzol^®^ Reagent. RNA concentration was quantified using the ND-2000 (NanoDrop Technologies) while RNA quality was determined by 5300 Bioanalyser (Agilent). According to the instructions (Illumina, San Diego, CA), RNA purification, reverse transcription, library construction and sequencing were performed at Shanghai Majorbio Bio-pharm Biotechnology Co., Ltd. (Shanghai, China). Raw data and clean data were obtained and quality controlled by fastp. Differential expression analysis was conducted using either DESeq2 or DEGseq. Further, Gene Ontology (GO) functional enrichment and Kyoto Encyclopedia of Genes and Genomes (KEGG) pathway analysis were carried out by Goatools and Python scipy, respectively (adjust P ≤0.01, |log2 (fold change)| >1).

### 2.15 Statistical analysis

Statistical analysis was performed using GraphPad Prism software 6.01 (GraphPad Software Inc., CA, USA) and SPSS 17.0 (IBM Corp., NY, USA). All experimental data were expressed as mean ± standard deviation of at least three independent experiments. Data were determined by one-way analysis of variance to compare the mean values of variables among the groups. Bonferroni’s test or Tukey’s *post hoc* test was used to identify the significance of pairwise comparison of mean values among the groups. Moreover, Spearman’s correlation analysis was performed to identify the correlations between microbiota and inflammatory indicators. *P ≤* 0.05 was considered to be statistically significant.

## 3 Results

### 3.1 Composition analysis of SPE

**Figure 2:**
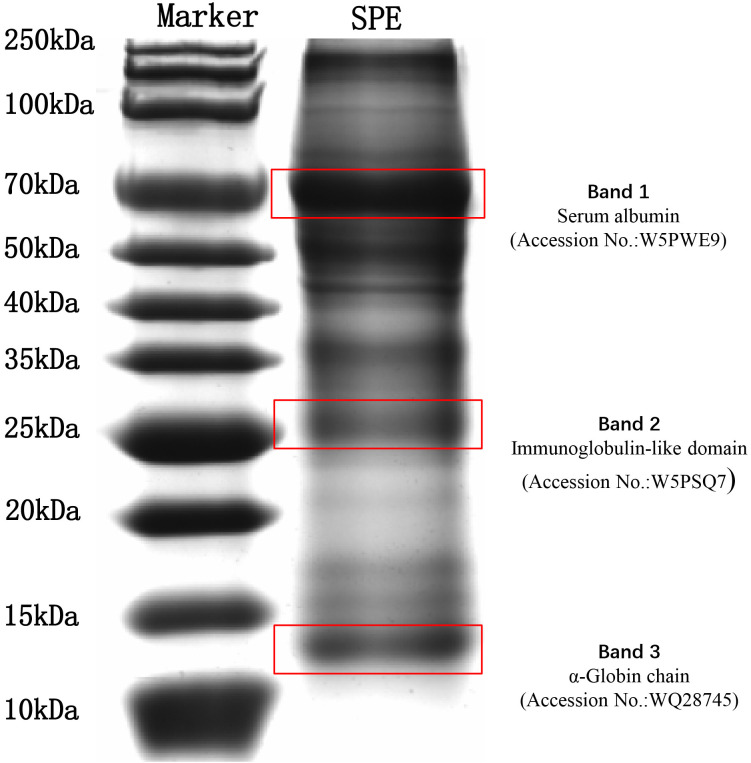
SDS-PAGE profiles and identification of proteins by mass spectrometry in SPE. Accession No. is the corresponding numbers of the proteins in UniProt database.

Previous study indicated the main proteins around 66.4 kDa were serum albumins, while the proteins around 27.0 kDa were immunoglobulin-like domains. The proteins around 14.3 kDa mainly belonged to the globulin family (11). To further confirm the composition of SPE, SDS-PAGE was performed to determine the main proteins and their molecular weight distribution. There were three main electrophoresis bands in the SDS-PAGE profiles of SPE, with molecular weight around 70.0 kDa, 25.0 kDa and 15.0 kDa (**Figure 2**). The albumins and immunoglobulin-like domains play important roles in immune system function and inflammation regulation (39). Apart from proteins, peptides and other bioactive compounds may also contribute to the bioactivity of placenta extract. Previous studies have indicated that peptides from goat and human placenta extracts have numerous bioactivities, including antioxidant and anti-inflammatory activities (40, 41). In addition, mass spectrometry identified 1,204 proteins (**Supplementary Table 1**) in the SPE. As shown in **Table 1**, the top 30 most abundant proteins and peptides related to proteins are listed, especially, Globin family profile domain is an important composition of the protein group.

**Table 1:** Top 30 proteins and relative peptides identified by LC-MS/MS in SPE samples.

Molecular weight	PG. PSMs	PG.UniprotIds	Peptide-length	Peptide	Protein
42kDa	1126	A0A836CS00	17	ISKQEYDESGPSIVHRK	Actin, cytoplasmic 1
60kDa	559	W5Q038	15	GFKGEGPEVDVNLPK	AHNAK nucleoprotein
50kDa	559	Q6DKR0	15	TKEVPVVVKFNRPFL	Serpin peptidase inhibitor, clade A
26kDa	493	W5PSQ7	32	RLLVVYPWTQRFFESFGDLSTPDAVMG	Globin family profile domain-containing protein
42kDa	482	W5PZK7	16	VTTAEREIVRDIKEKL	Actin alpha 2, smooth muscle
235kDa	344	A0A836AQA7	9	PAETPKPLG	Myosin light chain kinase
22kDa	299	A0A6P3T3Z3	11	SGPPVSELITK	Uncharacterized protein
57kDa	245	A0A836AL76;	18	GPIEEAVAKADKLAEEHS	ATP synthase subunit beta
50kDa	243	W5P0V0	18	SDKPDMAEIEKFDKSKLK	Thymosin beta-4
65kDa	218	W5PWE9	12	TCVADESAENCDK	Serum albumin
50kDa	214	A0A836CP05	26	APADVTSEKDVQAALTLAREKFGRVD	3-hydroxyacyl-CoA dehydrogenase type-2
31kDa	180	Q9XSY9	14	LPVKPTSSGSSEEK	Osteopontin
50kDa	179	W5PHA3	9	PPTRPTDKP	Tr-type G domain-containing protein
46kDa	169	A0A6P9FR52	16	DGKLPEVTKDVERTDG	Serpin H1
23kDa	162	A0A835ZW16	16	HEEAPGHRPTTNPNT	Cysteine and glycine-rich protein 1
21kDa	157	A0A6P3T3Y7	22	SETAPAAPAAPAPAEKTPVKK	Uncharacterized protein
40kDa	155	A0A6P3EDU1	15	SVGEGVTTVRPGDK	alcohol dehydrogenase
37kDa	155	A0A6P3TRD9	12	GIKEDTEEHHLR	Heterogeneous nuclear ribonucleoproteins A2/B1
53kDa	149	A0A6P3EK74	8	PLVDTHSK	Vimentin
91kDa	146	W5PRA1	14	PEYDEAGPSIVHRK	ACTG2
164kDa	141	A0A6P7DQB6	12	GVAEQLHNEGFK	Carbamoyl-phosphate synthase
92kDa	126	W5Q4E1	13	AAVEEEEDEKKPK	Endoplasmin
96kDa	125	A0A836D4G2	13	IEEVEVAPPKAHE	Alcohol dehydrogenase class-3
34kDa	106	W5NZJ5	13	MELIQDTSRPPAK	Sulfotransferase
59kDa	105	A0A8V6V2Q5	15	TDGKISEEADAKLKE	ATP synthase subunit alpha
61kDa	98	W5Q1L2	13	VGGTSDVEVNEKK	60 kDa heat shock protein, mitochondrial
280kDa	92	A0A6P7DLX7	16	SAPGPGPTDASKVLAK	Calponin-homology (CH) domain-containing
115kDa	91	W5NXB5	19	SAPAVKPGSKSTQAVPKAP	Microtubule-associated protein
16kDa	90	Q28745	10	TYFPHFDLSHGSAQVK	Alpha- globin chain
30kDa	85	W5NX51	15	LGEKAKPALEDLRQG	Apolipoprotein A-I

### 3.2 SPE supplementation improved the estrous cycles of letrozole-induced PCOS in rats

**Figure 3:**
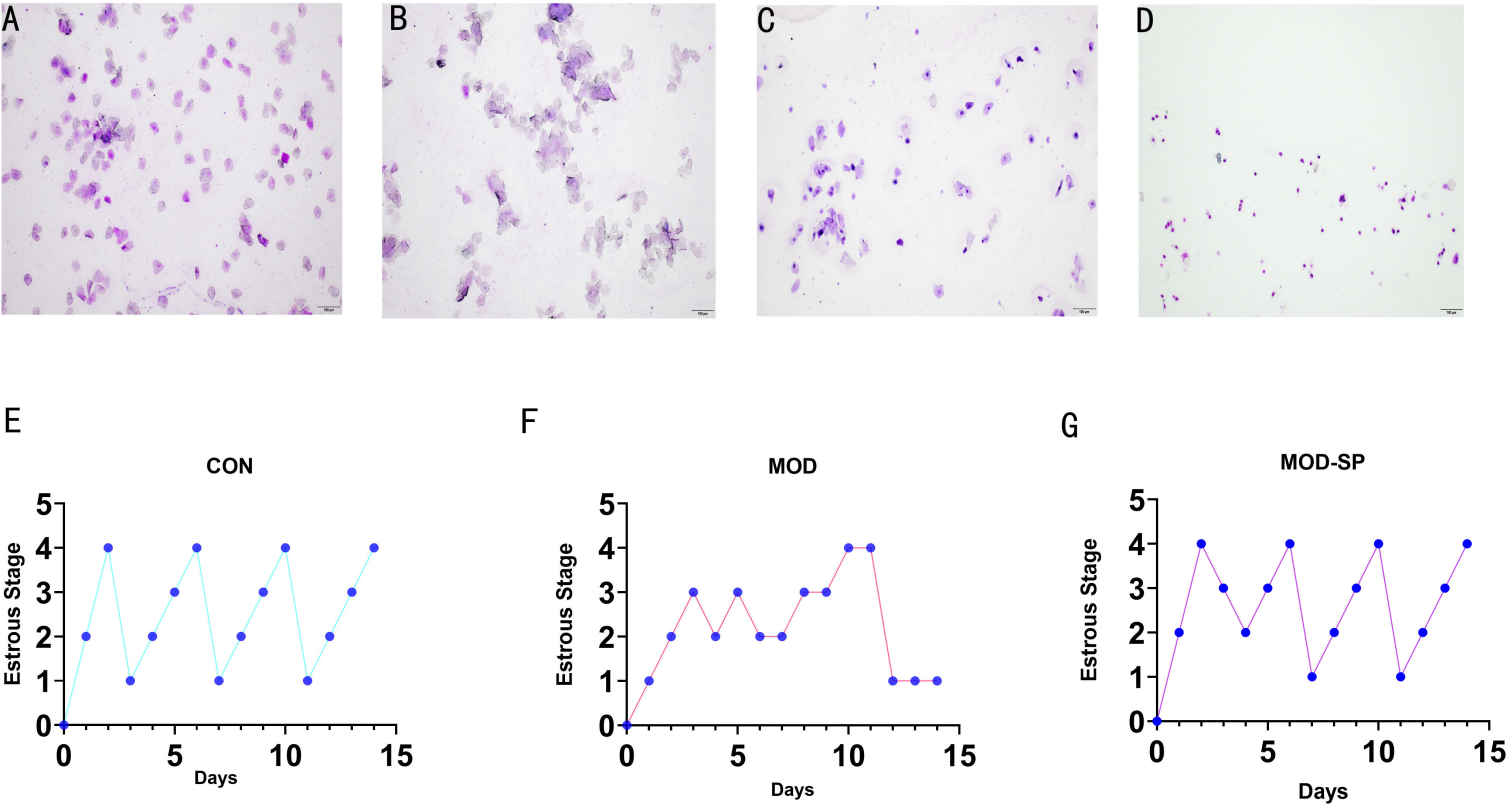
Estrous cycle changes in representative rats of each group. **(A)** Vaginal smears of proestrus stage. **(B)** Vaginal smears of estrus stage. **(C)** Vaginal smears of metestrus stage. **(D)** Vaginal smears of diestrus stage. **(E–G)** Representative estrous cycles of each group: 1, proestrus stage; 2, estrus stage; 3, metestrus stage; 4, diestrus stage. Original magnification (×100).

Wright–Giemsa staining was used to estimate the difference of estrous cycles in of each group. Rats in the CON groups exhibited regular estrous cycles of 4–5 days, comprising of proestrus, estrus, metestrus, and diestrus (**Figures 3A–E**). However, after 15 days of intervention, rats in the MOD experienced disrupted estrous cycles, indicating a prolonged diestrus state (**Figure 3F**). Intriguingly, after 15 days of dietary SPE intervention, regular estrous cycles were restored in 90% of the rats (**Figure 3G**), suggesting that dietary SPE can improve the disorder of estrous cycle in letrozole induced PCOS rats.

### 3.3 SPE mitigated ovarian pathological damage

**Figure 4:**
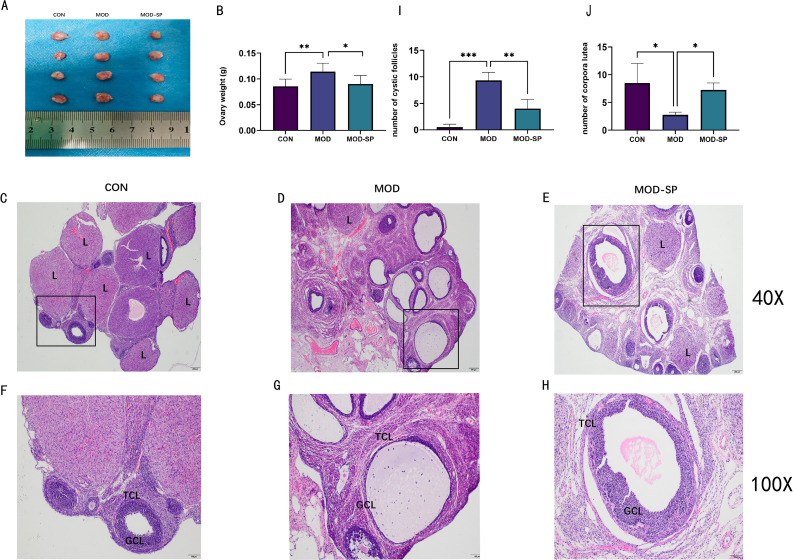
Effects of SPE on ovarian surface morphology and weight of each group. **(A)** Ovarian surface morphology. **(B)** Ovary weight. Effects of SPE on ovarian tissue morphology of each group with hematoxylin–eosin (H&E) staining: **(C)** CON, **(D)** MOD, **(E)** MOD-SP. The larger boxed areas in **(C–E)** (×40) are shown at higher magnification (×100) in **(F–H)**, respectively. **(I)** Numbers of cystic follicles. **(J)** Numbers of corpora lutea. TCL, theca cell layer; GCL, granular cell layer; L, luteum. Data were expressed as mean ± SD. *P < 0.05, **P < 0.01, ***P < 0.001.

Ovary weights in the MOD group were increased with a fairly smooth-white appearance, compared with those in CON group (*P* < 0.01) (**Figures 4A, B**). Interestingly, dietary SPE improved the ovarian morphology and the ovary weights in comparison with MOD group (*P* < 0.05) (**Figure 4B**). Histological analysis of ovarian tissues showed degenerated follicles and disrupted granulosa cells, thecal cells, antrum, and oocyte in PCOS rats compared with CON group. However, the ovarian tissues of MOD-SP group showed preserved follicles with normal granulosa cells, thecal cells, oocyte and large antrum (**Figures 4C–H**). In addition, the number of degenerated follicles was significantly higher (*P* < 0.001) in MOD group than those in the CON group, while those in the MOD-SP group was lower than those in the MOD group (*P* < 0.01) (**Figure 4I**). Similarly, the number of corpus luteum was significantly decreased (*P* < 0.05) in the MOD group compared to those in the CON group. However, after SPE intervention, those in the MOD-SP group were significantly increased (*P* < 0.05) in comparison with those in the MOD group (**Figure 4J**).

### 3.4 SPE improved morphometric changes in uterine tissue of rats with PCOS

**Figure 5:**
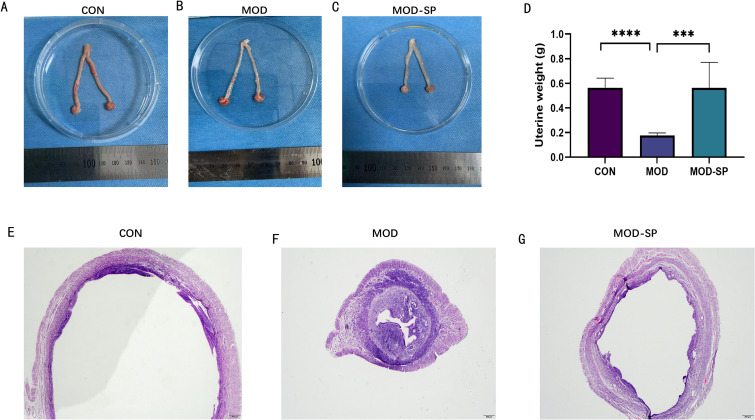
Effects of SPE on uterine surface morphology and weight of uterus in each group. **(A-C)** Uterine surface morphology. **(D)** Uterine weight. Effects of SPE on uterine tissue morphology of each group with hematoxylin–eosin (H&E) staining: **(E)** CON group, **(F)** MOD group, **(G)** MOD-SP group. Data were expressed as mean ± SD. ***P < 0.001. ****P < 0.0001.

In addition to ovarian dysfunction, the endometrium represents a critical independent target in PCOS, characterized by chronic unopposed estrogen exposure, insulin-driven hyperproliferation, and inflammation, resulting in markedly elevated risks of endometrial hyperplasia, adenocarcinoma, implantation failure, and adverse pregnancy outcomes (42). In this study, **Figure 5** displays morphology and photomicrographs of images of uterine tissue across experimental groups. Compared to the CON group, the MOD group had a smaller uterus size, while the MOD-SP group had a restored uterus size (**Figures 5A–C**). Moreover, uterine tissue weight in the MOD group was decreased with a smaller size appearance, compared with those in the CON group (*P* < 0.0001), while, dietary SPE administration reversed the uterine tissue weights compared with those in the MOD group (*P* < 0.001) (**Figure 5D**). Furthermore, at a pathological level, the endometrial wall thickness in the MOD group was greater than those in the CON group. SPE intervention reduced the thickness of the endometrial wall back to normal levels. (**Figures 5E–G**).

### 3.5 SPE suppressed body weight and ameliorated dyslipidemia

**Figure 6:**
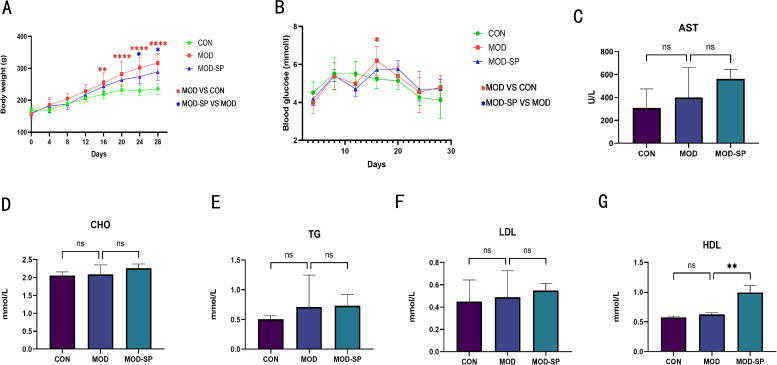
Effects of SPE on body weight (BW) and lipid metabolism indicators in each group. **(A)** Body weight growth curve. **(B)** Blood glucose levels. **(C)** Plasma glutamic oxaloacetic transaminase levels. **(D)** Plasma total cholesterol levels. **(E)** Plasma triglycerides levels. **(F)** Plasma low-density lipoprotein levels. **(G)** Plasma high-density lipoprotein levels. Data were expressed as mean ± SD. *P< 0.05, **P< 0.01, ****P < 0.0001, ns: *P*>0.05.

The body weight growth curve of rats in of each group was shown in **Figure 6A**. No significant differences in body weight (BW) were observed among the groups at the start of the intervention. After 16 days of the intervention, the BW in the MOD group were dramatically higher than those in the CON group (*P* < 0.01). However, after SPE treatment, the body weight growth curve in the MOD-SP group remained stable, especially after 24 days, BW exhibited a significantly decrease trends, compared to those in the MOD group (*P* < 0.05), suggesting that SPE relieved the BW in the PCOS model. There was no significant difference in the blood glucose levels among the three groups (**Figure 6B**).

To assess the effects of SPE on hepatic function and lipid metabolism in PCOS, glutamic oxaloacetic transaminase (AST), triglycerides (TG), total cholesterol (TC), low-density lipoprotein cholesterol (LDL-C), high-density lipoprotein cholesterol (HDL-C) were investigated, respectively (**Figures 6C–G**). The results showed no difference in the levels of AST, TG, TC, LDL-C in of each group (*P >*0.05). There was no difference in the levels of HDL-C between the CON and MOD groups (*P >*0.05). However, the level of HDL-C was higher in the MOD-SP group than that in the MOD group (*P* < 0.01), These results suggested that SPE intervention improved lipid metabolism and provided a protective effect against dyslipidemia.

### 3.6 SPE alleviated the plasma levels of sex steroid hormones in rats with PCOS

**Figure 7:**
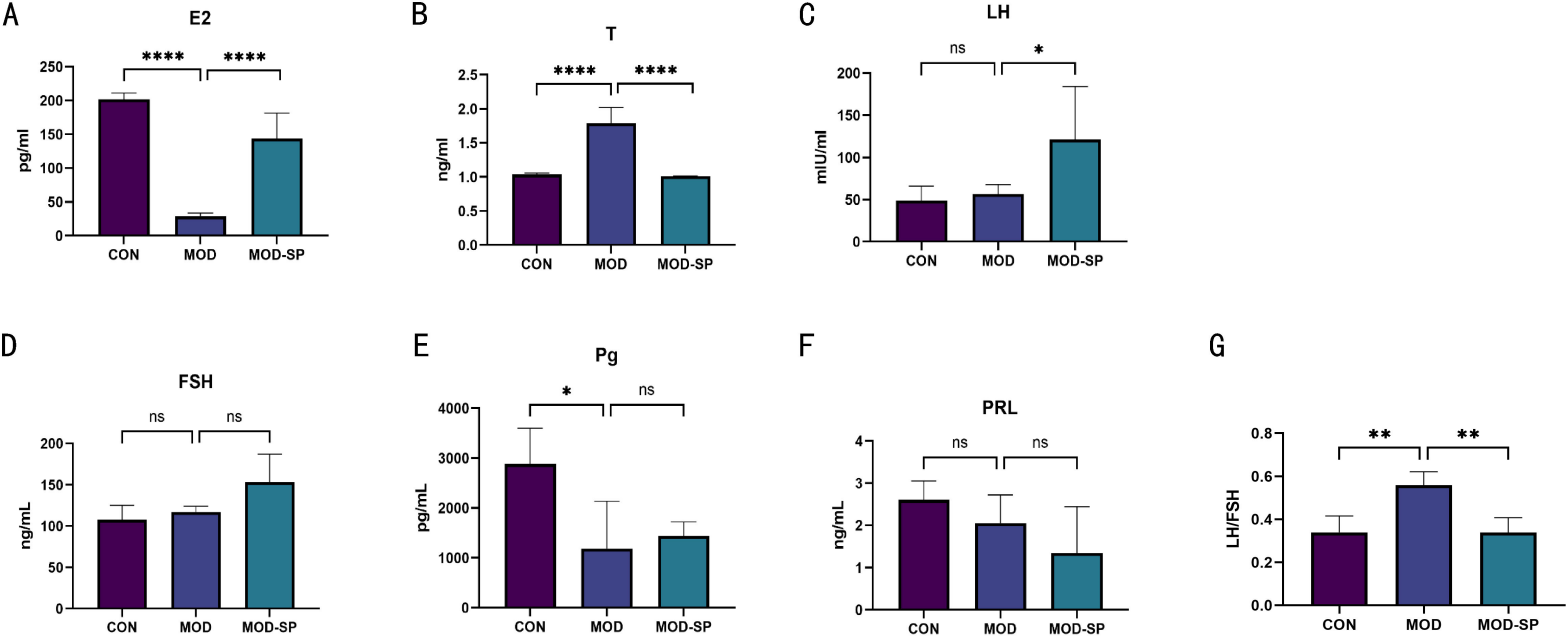
Effect of SPE on sex steroid hormones in PCOS- induced rats. **(A)** Plasma estradiol levels. **(B)** Plasma testosterone levels. **(C)** Plasma luteinizing hormone levels. **(D)** Plasma follicle-stimulating hormone levels. **(E)** Plasma progesterone levels. **(F)** Plasma prolactin levels. **(G)** LH/FSH ratio levels. Data were expressed as mean ± SD. *P< 0.05, **P< 0.01, ****P < 0.0001, ns: *P*>0.05.

It is well-known that PCOS rats typically exhibit disordered hormone levels (43). The results of our experiment indicated that the MOD group presented significantly diminished levels of estradiol (E2) and progesterone (Pg) (all *P* < 0.05), while levels of testosterone (T) and LH/FSH were significantly elevated (all *P* < 0.05) in comparison with the CON group. Compared to the MOD group, the MOD-SP group presented significantly lower levels of testosterone (T) and LH/FSH, while levels of estradiol (E2) and luteinizing (LH) levels were significantly higher (all *P* < 0.05) (**Figures 7A, B, E**). There was no difference in follicle-stimulating hormone (FSH), prolactin (PRL) levels among all groups (all *P >*0.05) (**Figures 7D, F**). These results demonstrate that SPE ameliorates reproductive hormone imbalances in PCOS.

### 3.7 SPE restored the levels of plasma inflammatory cytokines in PCOS

**Figure 8:**
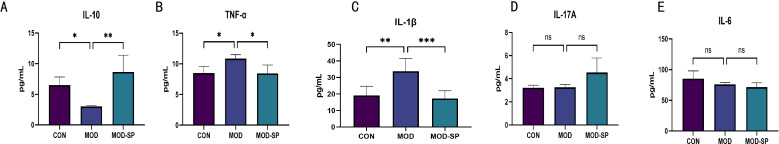
Effects of SPE on plasma inflammatory indicators in each group. **(A)** Plasma IL-10 level. **(B)** Plasma TNF-α level. **(C)** Plasma IL-1β level. **(D)** Plasma IL-17A level. **(E)** Plasma IL-6 level. Data were expressed as mean ± SD. *P< 0.05, **P< 0.01, ***P < 0.001. ns: *P*>0.05.

Studies have shown that inflammation is closely related to PCOS (12). Thus, we further analyzed the effect of SPE on inflammation in PCOS rats. Plasma level of interleukin-10 (IL-10) was significantly decreased (*P* < 0.05), while plasma level of tumor necrosis factor-alpha (TNF-α) and interleukin-1β (IL-1β) expression exerted significantly increased (all *P* < 0.01) in rats of MOD group, compared to the CON group. However, SPE administration significantly increased plasma level of IL-10 and decreased the plasma levels of TNF-α and IL-1β, compared with the MOD group (**Figures 8A–C**). Additionally, no significant difference was observed in the levels of interleukin-6 (IL-6) and interleukin-17A (IL-17A) among the groups (**Figures 8D, E**). Taken together, SPE treatment ameliorated the systemic inflammation in PCOS.

### 3.8 Significant enrichment in pathways after SPE intervention related to T cell receptor signaling and immune regulation

**Figure 9:**
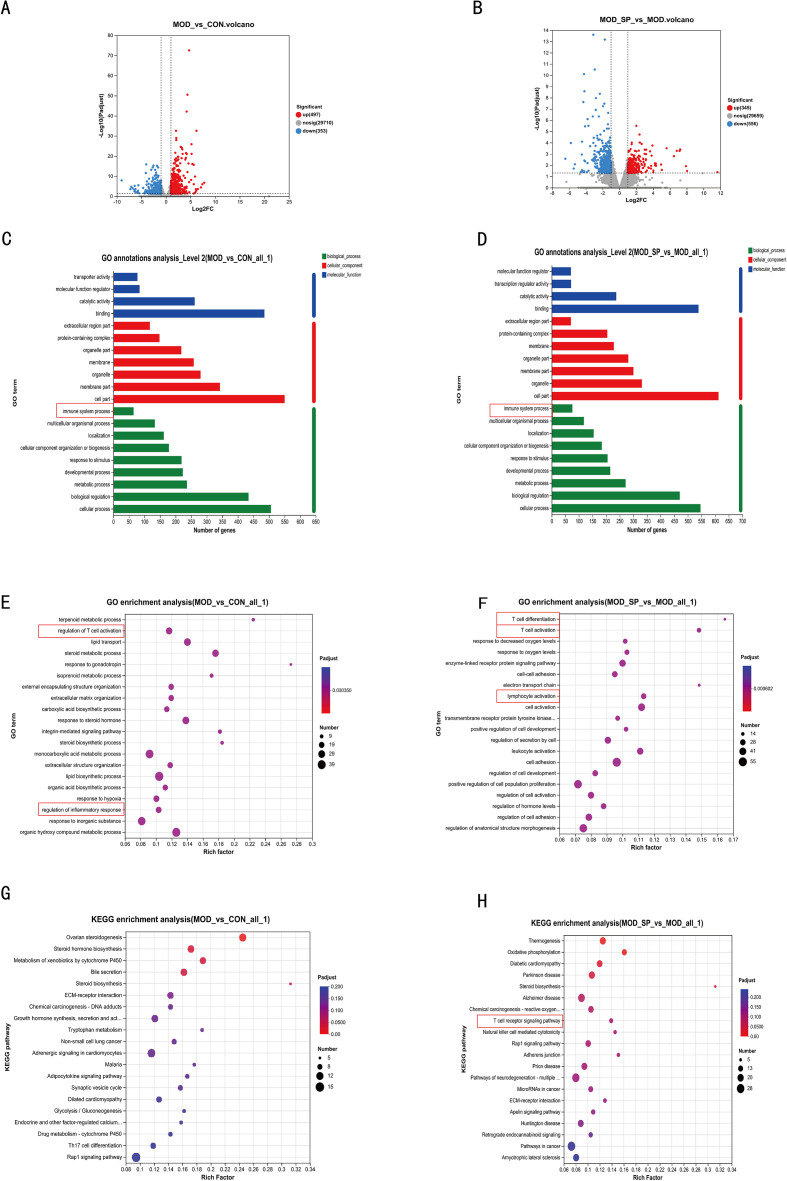
Transcriptomic sequencing. **(A)** Differential gene volcano map between control and model groups. **(B)** Differential gene volcano map (SPE intervention model vs model). **(C)** GO annotations analysis Level (model vs. control). **(D)** GO annotations analysis Level (SPE intervention model vs model). **(E)** GO enrichment analysis (model vs. control). **(F)** GO enrichment analysis (SP intervention model vs model). **(G)** KEGG enrichment analysis (model vs. control). **(H)** KEGG enrichment analysis (SPE intervention model vs model).

To further explore the anti-inflammation mechanism of SPE effectiveness in rats with PCOS, ovaries of CON, MOD and MOD-SP group were measured by transcriptomics. In the result, exactly 497 genes were upregulated, and 353 were down regulated in the model group compared with the control group (**Figure 9A**). Moreover, we found that 353 genes were upregulated and 556 were downregulated in the MOD-SP group compared to those in the MOD group (**Figure 9B**). Both Gene Ontology (GO) annotation of MOD-upregulated genes and SPE-upregulated genes demonstrated the enrichment in immune system processes (**Figures 9C, D**). Among these differentially expressed genes, GO enrichment analysis indicated regulation of T cell activation pathway and regulation of inflammatory responses pathway among the CON and MOD groups (**Figure 9E**). Importantly, the MOD and MOD-SP groups showed differences in the T cell differentiation, T cell activation and lymphocyte activation pathways (**Figure 9F**). KEGG enrichment analysis was performed on these different genes to show the top 20 pathways, including T cell receptor signaling pathway, according to the enrichment *P*-value (**Figures 9G, H**).

### 3.9 SPE alleviated the inflammation by modulating Tregs through estrogen receptor regulation in rats with PCOS

**Figure 10:**
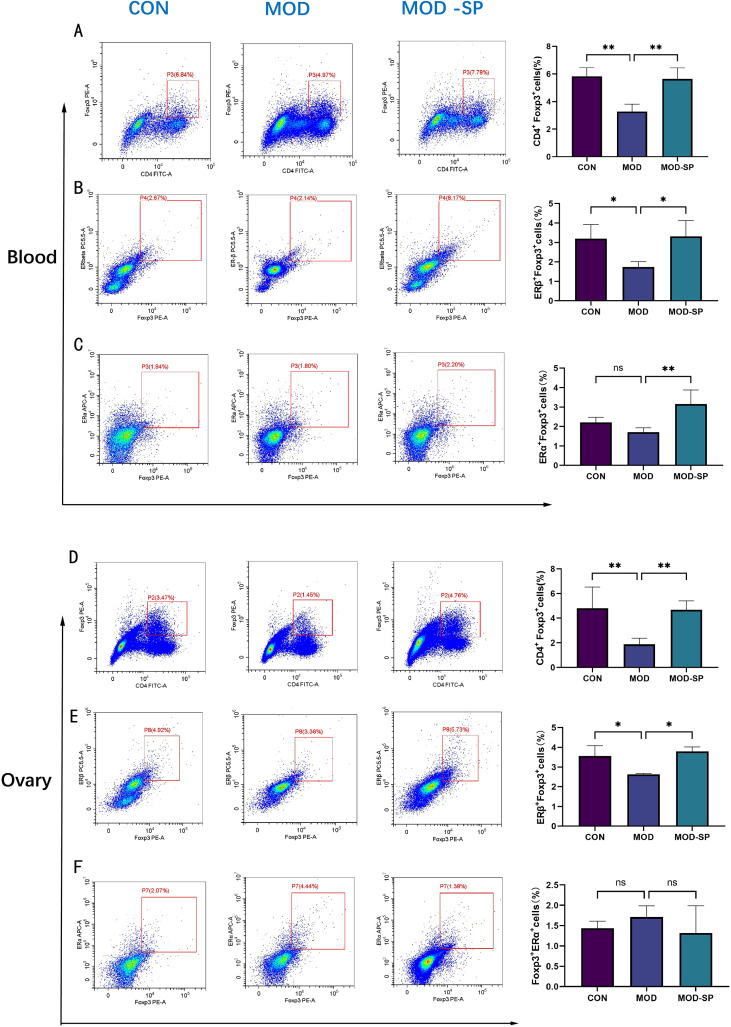
Effects of SPE on Treg cells, ERβ+ Treg cells and ERα+ Treg cells in the peripheral blood and ovarian tissue of rats with PCOS. **(A)** The percentage of Tregs within the total number of CD4+T lymphocytes in peripheral blood. **(B)** The percentage of ERβ+Tregs in peripheral blood. **(C)** The percentage of ERα+ Tregs in peripheral blood. **(D)** The percentage of Tregs within the total number of CD4+T lymphocytes in ovarian tissue. **(E)** The percentage of ERβ+Tregs in ovarian tissue. **(F)** The percentage of ERα+Tregs in ovarian tissue. Data are expressed as mean ± SD. *P < 0.05, **P < 0.01, ns: *P*>0.05.

Previous studies have shown that enhancing ER expression in CD4^+^ T lymphocytes promotes their differentiation into Treg (44, 45). To further explore the anti-inflammatory mechanisms of SPE, and the relationship between Treg cell and estrogen receptor (ER) expression of CD4^+^ T lymphocytes, we used flow cytometry to detect Treg cells and the expression levels of ERβ, ERα in peripheral blood and ovarian tissue. Compared to the CON group, the proportion of Tregs in the peripheral blood of the MOD group was significantly decreased (*P* < 0.01), indicating downregulation of Tregs in PCOS. Nevertheless, treatment with SPE significantly increased Tregs proportion compared to those in the MOD group (*P* < 0.01) (**Figure 10A**). In ovarian tissue, the trend of Treg proportion in of each group is same to peripheral blood (*P* < 0.01) (**Figure 10D**). The MOD group showed that the expression of ERβ in peripheral Treg cells was significantly lower than that in the CON group (*P* < 0.05). After SPE administration, the expression of ERβ in Treg cells was significantly elevated compared to MOD group (*P* < 0.05) (**Figure 10B**). Similarly, in ovarian tissue, the proportion of ERβ^+^ Tregs decreased in the MOD groups compared with the CON group (*P* < 0.05), while SPE treatment significantly increased proportion of ERβ^+^ Tregs in MOD-SP group compared to the MOD group (*P* < 0.05) (**Figure 10E**). In the peripheral blood, no significant difference in ERα^+^ Treg proportions was observed between CON group and MOD group (*P >*0.05). while SPE intervention dramatically elevated ERα^+^ Treg proportion compared with MOD group (*P* < 0.01) (**Figure 10C**). In the ovarian tissue, no significant differences in the proportion of ERα^+^ Tregs were observed among the groups (*P* > 0.05) (**Figure 10F**).

### 3.10 SPE promoted Tregs activation via the TGFβ1-Smad3 signaling pathway

**Figure 11:**
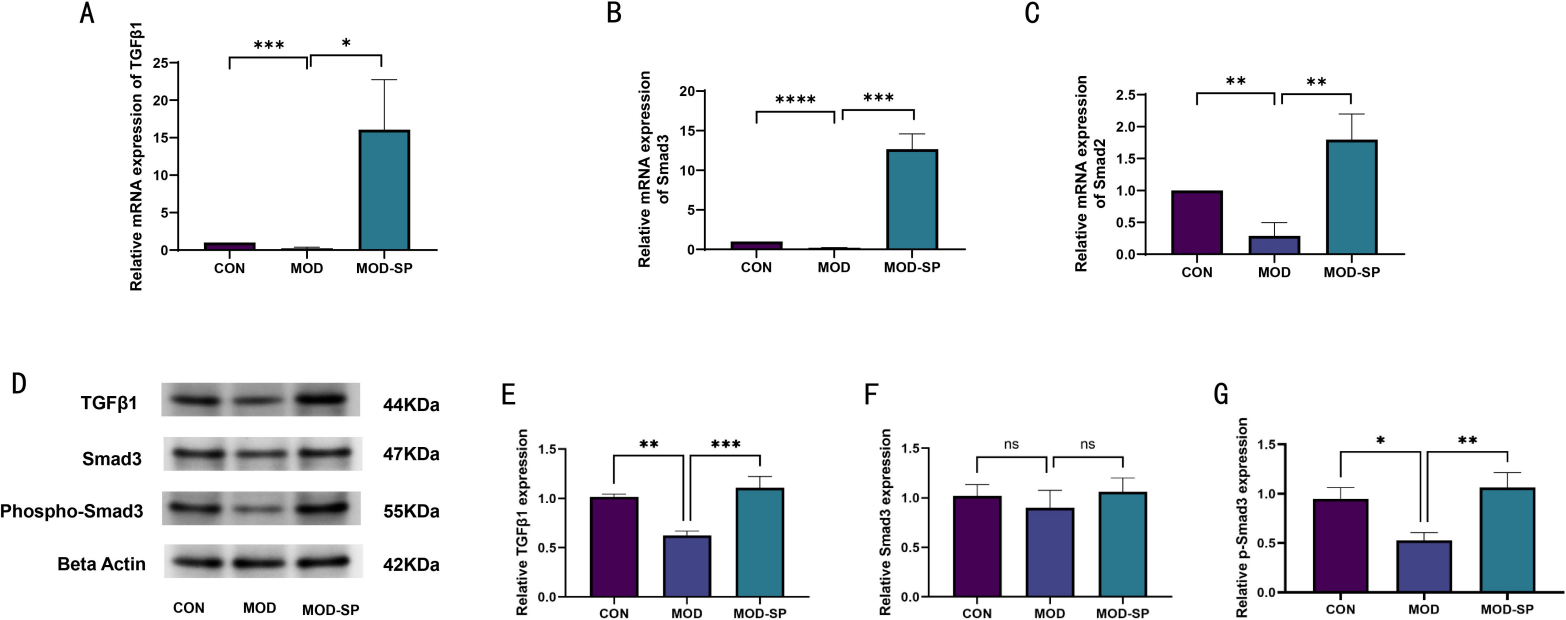
SPE alleviated the immune inflammatory response in PCOS through the TGFβ1/Smad3 signaling pathway. The mRNA levels of TGFβ1 **(A)**, Smad3 **(B)**, Smad2 **(C)** in the ovarian tissues. Representative western blot images and statistical results of TGFβ1 **(D, E)**, Smad3 **(D, F)**, and phospho-Smad3 (p-Smad3) **(D, G)** expressions of protein levels in the ovarian tissues. Data were expressed as mean ± SD. *P < 0.05, **P < 0.01, ***P < 0.001, ****P < 0.0001, ns: *P*>0.05.

Numerous studies have indicated that the TGFβ1-Smad3 signaling pathway plays pivotal role in the differentiation of CD4^+^ T cells into Tregs (46, 47). To explore the underlying mechanisms by which SPE ameliorates systemic inflammation in PCOS rats, qPCR analysis confirmed that SPE improved the expression of the target genes related to TGFβ1, Smad2, and Smad3 protein (**Figures 11A–C**). Finally, we verified that the expressions of crucial proteins, including TGFβ1 protein, Smad3/phosphorylated Smad3 protein, were consistent with the above results by western blot (**Figures 11D–G**). Overall, we demonstrated that SPE may regulate ovarian immune homeostasis mediated by activating Tregs through TGF-β1/Smad3 pathway.

### 3.11 Dietary SPE restored gut dysbiosis in PCOS

**Figure 12:**
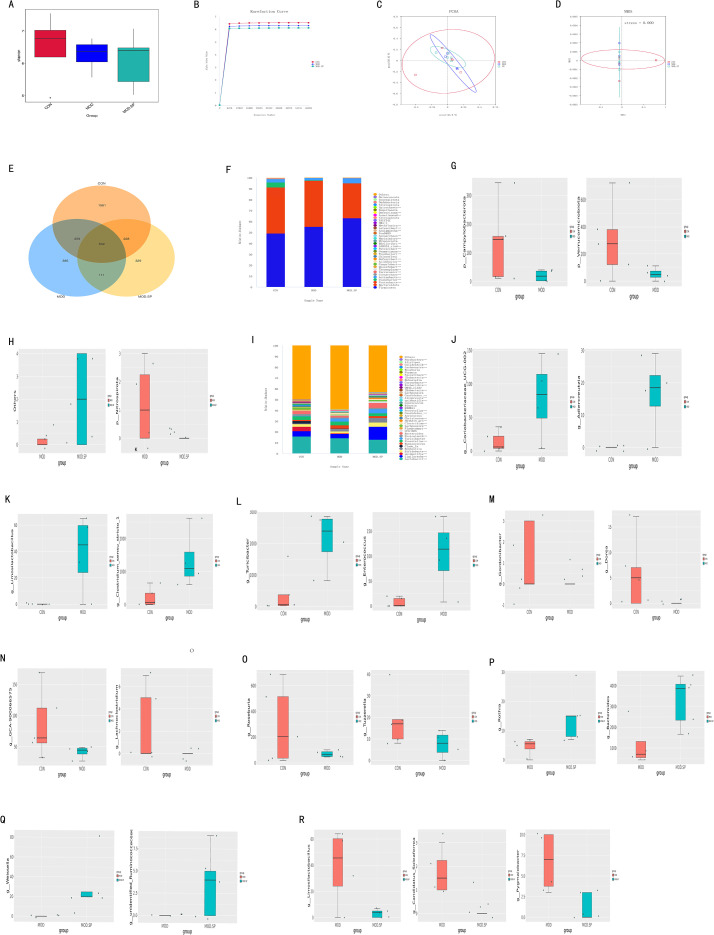
Gut microbial community in fecal samples of different groups. **(A)** Observed-species index. **(B)** Rarefaction Curve. **(C)** PCoA analysis. **(D)** NMDS analysis. **(E)** Venn diagram. **(F–H)** The phylum level. **(I–R)** The genus level. Values were given as mean ± SD. *P < 0.05.

Emerging evidence suggests that gut microbiota plays a critical role in the occurrence and progression of PCOS (30, 32). Therefore, we further investigated the changes in gut microbiota in rats following intervention with SPE. Fecal samples were analyzed using 16S rRNA high-throughput sequencing. As part of the α-diversity analysis, the “observed species” index and rarefaction curve were employed to evaluate bacterial community richness and diversity. The “observed species” index analysis revealed no significant changes in gut microbiota richness and diversity in the MOD group compared to the CON group. Similarly, no significant change was observed after SPE intervention (Wilcoxon rank-sum test) (*P* > 0.05) (**Figure 12A**). The rarefaction curve demonstrated the diversity of microbial species within each group. When the number of sequences increased to 6000, the rarefaction curve reached a plateau, indicating that the sequencing data were adequate (**Figure 12B**). Next, β-diversity of gut microbiota among different groups was evaluated using unweighted principal coordinate analysis (PCoA) and weighted distance matrix-based non-metric multidimensional scaling (NMDS). PCoA analysis showed no significant clustering differences in the overall gut microbiota composition between the MOD group and either the CON or MOD-SP groups (**Figure 12C**). NMDS analysis yielded similar results (**Figure 12D**). First, a Venn diagram revealed 632 core species shared among all groups, while the CON, MOD, and MOD-SP groups had 1561, 385, and 329 unique species, respectively (**Figure 12E**). Second, at the phylum level, *Firmicutes* and *Bacteroidetes* were the dominant bacteria across all groups (**Figure 12F**). Compared to the CON group, the proportion of *Campylobacterota* and *Verrucomicrobiota* significantly decreased in the MOD group (all *P* < 0.05) (**Figure 12G**). Compared to the MOD group, the proportion of other bacteria significantly increased in the MOD-SP group, while the proportion of *Nitrospirota* significantly decreased (*P* < 0.05) (**Figure 12H**). To further evaluate the impact of SPE on the gut microbiota structure at the genus level, we analyzed the top 40 species (**Figure 12I**). Compared to the CON group, the proportion of *Coriobacteriaceae_-_UCG_-_002*, *Adlercreutzia*, *Limosilactobacillus*, *Clostridium*, *Turicibacter*, and *Enterococcus* significantly increased in the MOD group (all *P* < 0.05) (**Figures 12J–L**), while the proportion of *Gordonibacter*, *Dorea*, *GCA_900066575*, *Lachnoclostridium*, *Roseburia*, and *Tuzzerella* significantly decreased (all *P* < 0.05) (**Figures 12M–O**). However, following SPE intervention, the proportion of *Rothia*, *Bacteroides*, *Weissella*, and *unidentified*_-_*Ruminococcaceae* increased (all *P* < 0.05) (**Figures 12P, Q**), while the proportion of *Limosilactobacillus*, *Candidatus_-_Soleaferrea*, and *Pygmaiobacter* decreased (all *P* < 0.05) (**Figure 12R**). Overall, our findings indicated that SPE significantly altered the abnormal genus composition ratio in PCOS by increasing *Rothia*, *Bacteroides, Weissella, Ruminococcaceae*, as well as reducing *Limosilactobacillus*, *Candidatus_-_Soleaferrea*, *Pygmaiobacter*.

## 4 Discussion

**Figure 13:**
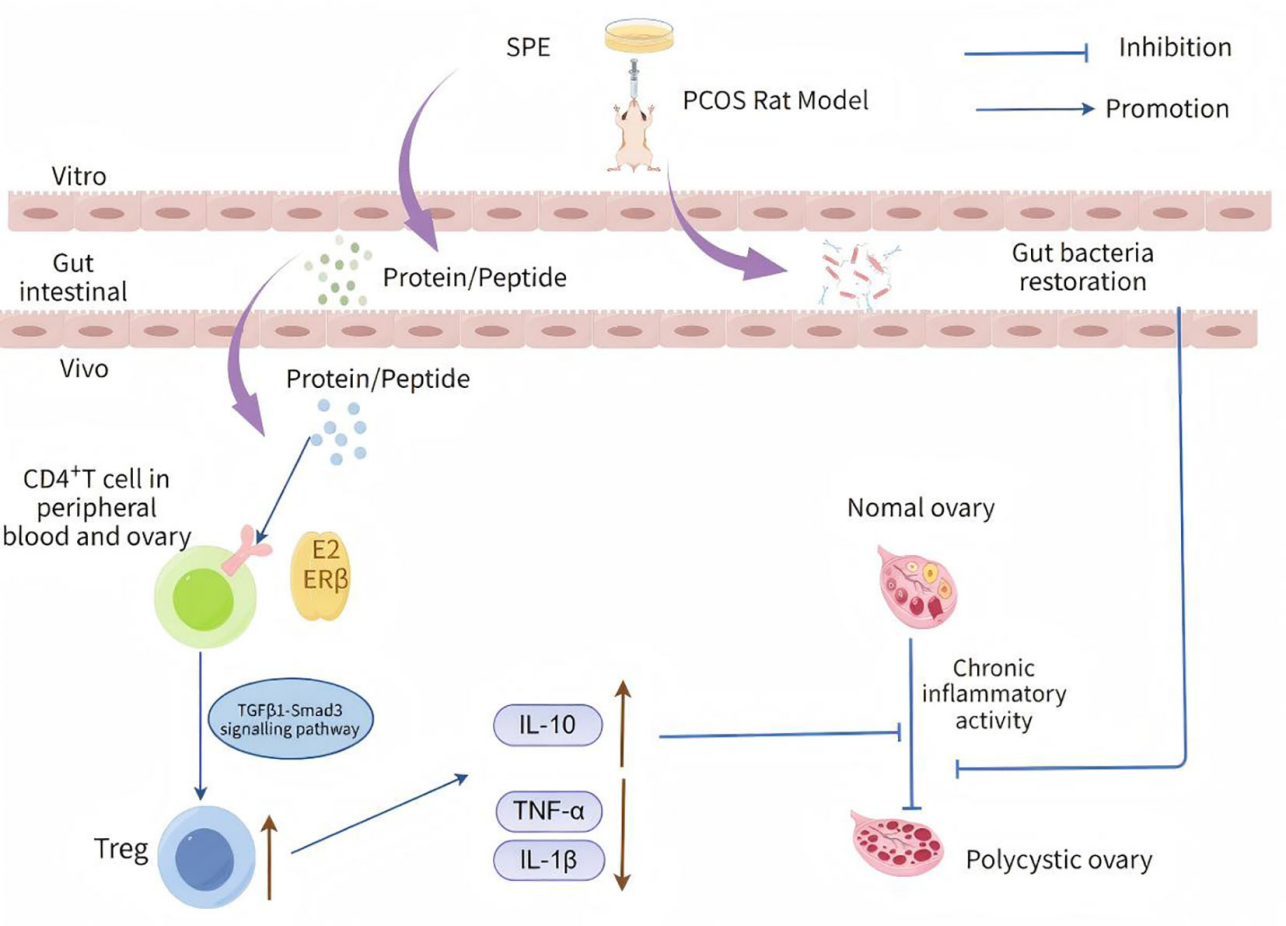
Schematic diagram of the mechanism in which SPE exerts therapeutic effects in PCOS rat model by suppressing inflammation via the ERβ/Treg axis, which is mediated by the TGF-β1/Smad3 signaling pathway, and restoring the balance of gut microbiota composition in PCOS rats.

In this study, we investigated the therapeutic potential of SPE, a traditional medicinal substance with documented anti-inflammatory, antioxidant, tissue-regenerative and endocrine-regulating properties, in a letrozole-induced PCOS rat model. Our results demonstrated that dietary supplementation with SPE significantly improved ovarian function, restored the estrous cycle, normalized sex steroid hormone levels, alleviated systemic inflammation and influenced the composition of the gut microbiota. However, the anti-inflammatory effects appear to be mediated through enhancing the stimulation of ERβ^+^ Treg via the TGFβ1-Smad3 signaling pathway (**Figure 13**). These findings offer new insights into immune inflammation regulation in PCOS and highlight the potential of SPE as a complementary intervention. .

The placenta is an important organ in mammals during pregnancy, functioning to provide hormones, nutrients and oxygen to the fetus (48). It contains various nutrients and bioactive components that are required for fetal growth and development, including hormones, amino acids, nucleic acids, proteins, vitamins, growth factors and cytokines (49). Therefore, the placenta is an important medication in traditional medicine. As recorded in the Compendium of Materia Medica “Bencao Gangmu” of Traditional Chinese Medicine (TCM), human placenta extract (HPE) are referred to as “Ziheche”, which are believed to nourish blood and qi, as well as the liver and kidney (50). However, limited availability and ethical issues prevent the widespread application of HPE. Some studies have demonstrated that animal placental extract have similar functions to those of the human placenta. Thus, the placentae of domestic animals such as sheep, camels, and cows can be used as an alternative to the human placenta. SPE is widely available in China due to the abundance of sheep farming resources and the particular focus on the clinical therapeutic potential of the active substances found in sheep placentas. Current researches on the application of placental extract include promoting anti-inflammatory, immune regulatory, wound healing and liver disease effects, as well as promoting hair growth and delaying ageing (51, 52). In our study, the results indicated SPE exhibited potent anti-inflammatory and immunomodulatory effects in PCOS. This is consistent with previous research on SPE (10).

PCOS is a chronic endocrine-metabolic disease characterized by hyperandrogenism and low estrogen levels (53). During the development and the progression of PCOS, abnormally increased T secretion suppresses the production of E2, and FSH (54–56). The LH/FSH ratio was regarded as a main biomarker of diagnosis in PCOS (57). The notably increased T level is usually considered as a marker of hyperandrogenism in PCOS (58). In this study, the results related to sex hormones similar to previous study, pre-abnormal elevations in plasma testosterone (T) and the LH/FSH ratio, as well as reduced plasma estradiol (E2) levels, were notably rectified by SPE intervention, demonstrating that SPE supplementation was capable of improving the homeostasis of sex steroid hormones in PCOS. Moreover, PCOS is a frequently occurring endocrine disorder among women of reproductive age, often accompanied by metabolic concerns including dyslipidemia, insulin resistance, and obesity (59, 60). Our research indicated that SPE not only ameliorated the abnormal weight gain but also elevated high-density lipoprotein cholesterol in PCOS rats. High-density lipoprotein cholesterol (HDL-C) has been considered as “good cholesterol,” and its protective role in cardiovascular health has been extensively studied (61). However, the underlying mechanism of ameliorating lipid metabolism of SPE is still necessary to be further investigated.

Systematic chronic inflammation (SCI) plays a pivotal role in the pathogenesis of various chronic disorders, including PCOS (19). Evidence of an imbalance between immune cells and inflammatory cytokines has been found in the serum, ovaries and organs of PCOS patients (62). The interplay between the inflammatory state, obesity, hyperandrogenism (HA) and insulin resistance (IR) leads to an increased metabolic imbalance and reproductive endocrine dysfunction in patients with PCOS (63). Furthermore, systematic chronic inflammation is a contributing factor to PCOS-related complications such as multi-organ dysfunction, non-alcoholic fatty liver disease and cardiovascular disease (14, 19). Chronic systemic inflammation observed in PCOS patients is linked to an imbalance between pro-inflammatory and anti-inflammatory intrinsic mechanisms. As well as cytokines play a pivotal role in the pathogenesis of PCOS (64). In our study, we found that SPE alleviated systemic inflammation by suppressing the pro-inflammatory cytokine TNF-α, IL-1β and increasing the anti-inflammatory cytokine IL-10, suggesting an anti-inflammatory role for inexpensive SPE administration in PCOS. Consistent with the results of this experiment, previous studies showed that TNF-αsystem might contribute to the pathogenesis of HA, Ob, and IR in PCOS (65). Decrease in IL-10 may contribute to ovarian dysfunction and metabolic abnormalities by impairing anti-inflammatory responses, thereby promoting immune dysregulation and chronic inflammation (66). In addition, IL-1β is a key mediator of the NLRP3 inflammasome and systemic inflammatory cascades (13, 63). Accumulating evidences on the critical role of Tregs in the pathogenesis of many chronic diseases including PCOS (67), we speculate that the anti-inflammatory effect of SPE may attribute to the activation of Treg and differentiation of Tregs. In this study, the flow cytometry results indirectly verified this finding. Additionally, studies indicated that inflammatory processes can precipitate dysregulation across multiple systems, including metabolic, gut microbiota, and endocrine pathways (68). Conversely, perturbations in these systems may engender chronic inflammation, which in turn exacerbates the underlying dysregulations, thereby perpetuating a self-reinforcing vicious cycle. Furthermore, an isolated perspective is inadequate for elucidating the etiology of many chronic inflammatory diseases, including PCOS.

Given the anti-inflammatory effect of SPE in PCOS Rats, we performed transcriptomics and found differentially expressed genes in the ovarian tissue of PCOS rats. By comparing with the CON and MOD group, we identified a series of differentially expressed genes related to inflammation and regulation, which were further validated through bioinformatics analysis and flow cytometry validation. In total, we detected 30,560 genes in the ovarian tissue, with 497 genes upregulated and 353 genes downregulated in the MOD group versus the CON group. Similarly, 345 genes upregulated and 556 genes downregulated in the MOD-SP group versus the MOD group.

Trough KEGG and GO analysis of these different genes, we found that they were significantly enriched in the T cell receptor signaling pathways, and T cell-related signaling pathways. Furthermore, T cell-related pathways and their associated signaling cascades underpin the chronic inflammatory milieu in PCOS, in which adaptive immunity drives ovarian dysfunction through imbalanced lymphocyte polarization and cytokine dysregulation. In patients with PCOS, peripheral blood mononuclear cells exhibit increased Th17 cell frequencies alongside decreased Tregs. This creates a pro-inflammatory loop that exacerbates hyperandrogenism and insulin resistance via IL-17-mediated granulosa cell apoptosis and theca cell proliferation (69). In this study, the enrichment of the T cell receptor signaling pathways suggests that these pathways may play a key regulatory role in inflammation and immune regulatory development in PCOS rats. Studies have demonstrated the critical role of the T cell receptor (TCR) signaling pathway in adaptive immune regulation, suggesting that its dysregulation may lead to exacerbated inflammation and impaired tolerance induction (70). Our studies have demonstrated that T cell dysregulation plays a role in the development of PCOS. Overactivation of T cell receptor signaling pathways can lead to follicular developmental disorders by promoting ovarian cell proliferation and inhibiting apoptosis, which ultimately affects ovarian function pathological progression in PCOS. Studies demonstrated the T cell receptor signaling pathways plays a key role in the pathogenesis of PCOS, particularly in ovarian endocrinology and the regulation of follicular development (16). However, the exact role in the pathological development of ovarian follicular cystic in the PCOS has not been thoroughly investigated.

Present studies demonstrate Tregs restrain self-reactive immune responses and excessive inflammation in humans and mice (71). In addition to these immunoregulatory functions, Tregs have roles in tissue repair and homeostasis (72, 73). Both immunoregulatory and tissue repair functions render the Treg cell an potential therapies against chronic low-grade inflammation and abnormal immune responses (74). Combining these findings with ovarian transcriptomic, our results indicated that T-cell-associated signaling pathways were linked to the anti-inflammatory effect of PCOS. Further results of flow cytometry conformed anti-inflammatory effect in PCOS in which proportion of Tregs in PCOS rats decreased compared with the CON group. A study has also shown that the number of Treg cells in peripheral blood of PCOS patients decreased (75). However, SPE administration significantly increased the proportion of Tregs in the peripheral blood and ovaries of PCOS rats. Thus, these results demonstrated that SPE exerts anti-inflammatory effects by promoting the proportion of Treg in peripheral blood and ovarian tissue, as with previous studies confirming anti-inflammatory effect of SPE in various diseases. This highlights SPE as a promising adjunctive therapeutic agent with anti-inflammatory and immune-modulating effect in PCOS related to chronic inflammation via Tregs.

Estrogen, as a pivotal sex hormone, not only play a pivotal role in reproductive system functions but also profoundly modulates immune homeostasis and inflammatory responses through its nuclear receptors—estrogen receptor α (ERα) and estrogen receptor β (ERβ) (26). Within CD4^+^ T cells, the expression and signaling pathways of ERα and ERβ constitute the core components of the estrogen-mediated immune regulatory network, particularly in their interactions with Tregs. This interplay exerts a critical influence on the maintenance of peripheral immune tolerance, suppression of autoimmune diseases, and modulation of microenvironments in infections and tumors (76). The role of ERβ in CD4^+^T cells is predominantly immunosuppressive, particularly through enhancement of Treg cell function to sustain immune tolerance. ERβ knockout or signaling blockade impairs TGF-β-dependent Treg differentiation, thereby disrupting peripheral tolerance mechanisms and precipitating functional loss of Tregs in the aging female reproductive tract (44). Specifically, estradiol (E2) activates the Foxp3 transcription factor via ERβ, thereby promoting the induced generation and suppressive activity of Treg cells (28). In model of pneumonia, the E2/ERβ axis specifically modulates the suppressive effects of Tregs on macrophages, facilitating the resolution of pulmonary inflammation (28). In our study, compared to the CON group, the level of serum E2 decreased in PCOS model rats, but which was elevated by SPE administration. In addition, our flow cytometry results indicated that ERβ^+^ Tregs in the peripheral blood and ovarian tissue of PCOS rats was increased by SPE intervention. Moreover, the proportion of ERα^+^ receptors on Tregs in peripheral blood showed significant change, but ovarian tissue showed no significant change. Therefore, our data support the E2-ERβ axis enhances Tregs, thereby promoting Tregs-mediated anti-inflammation and maintaining immune homeostasis. In addition, TGF-β1/Smad signaling pathway is critical for the activation of Tregs to inhibit inflammatory responses (77, 78). Consistently, we demonstrated that the inhibition of inflammation of SPE may attributed to TGFβ1-Smad2/Smad3 pathway. This sequential signaling cascade drives Treg stimulation, ultimately leading to suppressing ovarian inflammation and restoring follicular development.

Previous studies have shown that the gut microbiota, as the largest microbial ecosystem in the human body, plays a key role in maintaining immune homeostasis, metabolic balance, and inflammatory regulation (79). Under normal circumstances, the gut microbiota produces metabolites such as short-chain fatty acids (SCFAs) including butyrate and propionate, to maintain tight junctions in epithelial cells and prevent harmful substances from entering the bloodstream (80). However, in a state of dysbiosis, beneficial bacteria, such as *Bacteroides* and *Clostridium* species decrease, while pathogenic bacteria, such as certain *Proteobacteria* increase, leading to increased intestinal permeability, or “leaky gut”. This allows endotoxins, such as lipopolysaccharides (LPS) produced by Gram-negative bacteria, to enter the bloodstream, and cause endotoxemia (81). LPS activates Toll-like receptors (TLRs), stimulating monocytes, macrophages, and dendritic cells to release pro-inflammatory cytokines such as TNF-α, IL-6, and IL-1β, thereby triggering systemic inflammation (82). We observed that abundances of *Firmicutes*, *Bacteroidetes*, and *Proteobacteria* were the most dominant in all groups in phylum level, which was consistence with previous studies (83). Importantly, SPE supplementation enriched beneficial bacterial *genera* such as *Rothia*, *Bacteroides* and *Weissella*, whereas reducing *Limosilactobacillus*, *Clostridium*, and *Enterococcus*. Although the functional roles of some of these taxa in PCOS are still being elucidated, *Bacteroides* species are known producers of short-chain fatty acids (SCFAs), particularly butyrate and propionate, which have been shown to enhance Treg differentiation and exert anti-inflammatory effects in the gut systemically (84). Conversely, reductions in *Limosilactobacillus* genus associated with dysbiosis under certain condition may reflect a shift toward microbial eubiosis. These microbial shifts suggest that SPE may influence host physiology directly or indirectly through the gut-ovary axis, a communication network gaining increasing attention in reproductive medicine. Indeed, emerging evidence indicates that gut dysbiosis contributed to PCOS progression via increased intestinal permeability, endotoxemia, and systemic inflammation (85). By reshaping the gut microbiome toward a more favorable composition, SPE may reduce metabolic endotoxemia and dampen systemic inflammation, thereby creating a permissive environment for ovarian recovery. Tregs, acting as the “brakes” of immune system, play a pivotal role in regulating inflammatory balance. They exert anti-inflammatory effects by producing anti-inflammatory cytokines, such as IL-10 and TGF-β, which suppress excessive immune responses and maintain immune tolerance (86, 87). The primary pathway through which gut microbiota dysbiosis impacts regulation of Treg is metabolite-mediated. Short-chain fatty acids (SCFAs), produced by beneficial bacteria, promote Treg differentiation. However, dysbiosis reduced SCFA levels, leading to Treg dysfunction and Th17/Treg imbalance (88, 89). Therefore, SPE may improve ovarian pathological abnormality associated with PCOS by enhancing Treg function through restoring gut microbiota composition and its metabolites, thereby alleviating the inflammatory microenvironment. However, the exact role by which SPE influenced PCOS pathology via the gut microbiota need to be further investigated.

Traditional Chinese Medicine (TCM) represents a vast pharmacological reservoir, with numerous herbal compounds exhibiting multi-target therapeutic effects (90). Notably, natural products such as resveratrol, berberine, curcumin, and artemisinin have demonstrated considerable pharmacological potential in modulating oxidative stress, insulin sensitivity, and inflammatory response (91). Similarly, placenta extract (PE), including SPE, is a well-known substance in Chinese traditional medicine that has beneficial pharmacological effects, including anti-inflammation, im-munomodulation, antioxidant activity and endocrine regulation, which are beneficial to conditions including PCOS. However, due to the complex composition of SPE, identifying the specific agents in SPE associated with anti-inflammatory and immunomodulatory effects is needed. Thus, leveraging new artificial intelligence (AI)-driven approaches to identify and characterize novel bioactive agents from the SPE may be a potential strategy.

Although the present study demonstrates the protective effect of SPE in a PCOS rat model, further experiments are required to refine and support our existing findings. Firstly, although the use of letrozole-induced PCOS model, while widely accepted, does not fully recapitulate the heterogeneous etiology of human PCOS, which includes genetic, environmental, and lifestyle factors, we will use animal models of PCOS induced by high-fat diets and other factors to further validate the protective effects and mechanisms of SPE in PCOS. Secondly, given the complexity of the SPE components, including a wide variety of proteins, peptides, growth factors, lipids and nucleic acids, it is challenging to attribute effects to specific molecules without conducting further isolation studies. In this study, although we initially employed a simple combination of LC-MS/MS and SDS-PAGE to identify target proteins related to immune inflammation. Nevertheless, this combined approach has limitation. Further study, we will employ the proteomics strategy, in conjunction with additional advanced analytical techniques, as well as network pharmacology and molecular docking, to achieve more precise characterization of the protein composition and biological functionalities of SPE, aiming to develop potential therapies for various diseases, including PCOS. Additionally, further studies should incorporate multi-batch LC-MS/MS metabolomics analysis into routine quality control protocols in order to quantify chemical variations between batches and define safety thresholds for their biological effects. The results of this study provide preliminary functional evidence for this standardization process. Subsequent work will focus on developing a more comprehensive correlation model between chemicals and efficacy for SPE, ensuring its reproducibility in clinical applications. Thirdly, our sample size is modest, and larger validation studies would strengthen statistical power. To address this issue, further studies should prioritize larger validation cohorts to strengthen the robustness, replicability, and broader applicability of the observed results. Fourth, the absence of comparisons with conventional therapies, such as metformin or glucagon-like peptide-1 receptor agonists (GLP-1 receptor agonists), may restrict the generalizability of our findings and ability to assess their comparative efficacy. To mitigate this, future validation studies employing alternative PCOS models should include these conventional treatments, such as metformin or GLP-1 receptor agonists, for comparison. This would enable a more thorough evaluation of the therapeutic effects, relative advantages and potential synergistic interactions of SPE. Fifth, through flow cytometry experiments, we validated the relationship between E2/ER axe and Tregs, as with previous studies. However, the use of estrogen receptor (ER) knockout animal models is required for further verification of this relationship and its underlying mechanisms. Sixth, to gain a more comprehensive understanding of the effect of different doses of SPE and different batches of SPE on PCOS, further study will systematically evaluate the dose-response relationship of multiple SPE doses in PCOS model rats to support translational research, and evaluate the immunomodulatory efficacy of SPE from multiple independent production batches in PCOS models, in order to validate the reproducibility and robustness of the treatment outcomes. Seventh, we acknowledge that systemic inflammation is a complex, multi-pathway process involving a diverse network of mediators. A limited panel of cytokines, even including IL-1β, TNF-α may not fully specific inflammatory phenotypes. To establish a more definitive and comprehensive evaluation system for systemic inflammation, future study will include a broader spectrum of inflammatory markers, including acute-phase reactants (e.g., C-reactive protein [CRP], Serum Amyloid A [SAA]), and pro-inflammatory cytokines (e.g., IL-18), and chemokines (e.g., MCP-1). Although our findings demonstrated that SPE improved the gut microbiota composition, further investigation is needed to assess gut barrier integrity and the levels of LPS and SCFAs related to microbiota changes in PCOS. Finally, long-term safety and dose-response of SPE need further investigation before any translational applications.

## 5 Conclusion

The present study demonstrated that SPE mitigate ovarian pathology in PCOS rat by suppressing inflammation via the ERβ)/Treg axis, which is mediated by TGF-β1/Smad3 signaling pathway. Additionally, SPE restored the balance of gut microbiota composition in PCOS rats, which may potentially serve as an inexpensive intervention for the control of PCOS patients.

## Data Availability

The datasets presented in this study can be found in online repositories. The names of the repository/repositories and accession number(s) can be found below: https://www.ncbi.nlm.nih.gov/, PRJNA1370456 and PRJNA1370467.
